# Anti-Inflammatory and Active Biological Properties of the Plant-Derived Bioactive Compounds Luteolin and Luteolin 7-Glucoside

**DOI:** 10.3390/nu14061155

**Published:** 2022-03-09

**Authors:** Sabrina Caporali, Alessandro De Stefano, Cinzia Calabrese, Alfredo Giovannelli, Massimo Pieri, Isabella Savini, Manfredi Tesauro, Sergio Bernardini, Marilena Minieri, Alessandro Terrinoni

**Affiliations:** 1 Department of Industrial Engineering, University of Rome Tor Vergata, 00133 Rome, Italy; sabrina.caporali93@gmail.com; 2 Centre of Space Biomedicine, Department of Systems Medicine, University of Rome Tor Vergata, 00133 Rome, Italy; alessandrodestefano6@virgilio.it (A.D.S.); mtesauro@tiscali.it (M.T.); 3 Department of Experimental Medicine, University of Rome Tor Vergata, Via Montpellier, 1, 00133 Rome, Italy; cinzia_cal@libero.it (C.C.); alfredo.giovannelli@gmail.com (A.G.); massimo.pieri@uniroma2.it (M.P.); savini@uniroma2.it (I.S.); bernardini@med.uniroma2.it (S.B.)

**Keywords:** flavonoids, flavones, luteolin, luteolin-7-O-glucoside (LUT-7G), inflammation, glucose metabolism, endothelial inflammation

## Abstract

Flavonoids are interesting molecules synthetized by plants. They can be found abundantly in seeds and fruits, determining the color, flavor, and other organoleptic characteristics, as well as contributing to important nutritional aspects. Beyond these characteristics, due to their biochemical properties and characteristics, they can be considered bioactive compounds. Several interesting studies have demonstrated their biological activity in different cellular and physiological processes in high-order organisms including humans. The flavonoid molecular structure confers the capability of reacting with and neutralizing reactive oxygen species (ROS), behaving as scavengers in all processes generating this class of molecules, such as UV irradiation, a process widely present in plant physiology. Importantly, the recent scientific literature has demonstrated that flavonoids, in human physiology, are active compounds acting not only as scavengers but also with the important role of counteracting the inflammation process. Among the wide variety of flavonoid molecules, significant results have been shown by investigating the role of the flavones luteolin and luteolin-7-O-glucoside (LUT-7G). For these compounds, experimental results demonstrated an interesting anti-inflammatory action, both in vitro and in vivo, in the interaction with JAK/STAT3, NF-κB, and other pathways described in this review. We also describe the effects in metabolic pathways connected with inflammation, such as cellular glycolysis, diabetes, lipid peroxidation, and effects in cancer cells. Moreover, the inhibition of inflammatory pathway in endothelial tissue, as well as the NLRP3 inflammasome assembly, demonstrates a key role in the progression of such phenomena. Since these micronutrient molecules can be obtained from food, their biochemical properties open new perspectives with respect to the long-term health status of healthy individuals, as well as their use as a coadjutant treatment in specific diseases.

## 1. Flavonoids in Nature and Chemical Structure

Flavonoids are polyphenolic secondary metabolites synthetized by plants, being abundantly found in seeds and fruits. These compounds play a crucial role in protecting plants from oxidative stress and microbial infection [1,2]. Moreover, flavonoids confer resistance to heat, freezing, drought, and UV irradiation. They are also responsible for the color and the aroma of flowers and fruits, which are the main characteristics for attracting pollinator insects [2].

Indeed, especially in occidental countries, the wide availability of foods rich in sugar and fat increases the onset of chronic illnesses such as metabolic, cardiovascular, and neurodegenerative disorders [3]. Besides their relevance in plants, flavonoids can potentially even improve human health [4,5].

In this setting, the daily intake of flavonoids through vegetables and fruits (see Table 1) could ameliorate the issues affecting general state of health, as these compounds are able to affect the metabolism and exert antioxidant, anti-inflammatory [6], and neuroprotective actions [7]. Thus far, over 10,000 flavonoids have been isolated and identified [8].

In general, flavonoids are phenylbenzo-pyrone derivates (Figure 1) characterized by a basic skeleton of 15 carbon atoms (C6–C3–C6) consisting of two aromatic rings (A and B) linked by a three-carbon chain to an oxygenated heterocycle (ring C). Flavonoids can be divided into different subclasses; flavones, flavonols, flavan-3-ols, flavanones, isoflavones, and anthocyanins. This classification depends on the hydroxylation and saturation patterns of the oxygenated heterocycle C.

Flavonoids are endorsed as having antioxidant and anti-inflammatory properties and even pharmacological potential as antitumoral [10], antimicrobial, antiviral, and antiangiogenic compounds [11,12], in essence due to their chemical structure.

In fact, flavonol consumption (mainly quercetin and kaempferol) has been associated with protective effects on the cardiovascular system [13,14], while anthocyanins seem to decrease the risk of acute myocardial infarction [15]. Instead, flavanones such as naringenin and hesperetin have been shown to be able to prevent the activity of free radicals [16]. Isoflavones, mostly daidzein and genistein, are structurally similar to mammalian estrogens, suggesting their ability to bind estrogen receptors [17]. The group of flavones includes luteolin (3′,4′,5,7-tetrahydroxy flavone) and apigenin (4′,5,7-tri-hydroxy-flavone) that, like the other flavonoids (see Table 2), exert different biological activities due to modifications in the chemical structure as hydroxylation, O-/C-glycosylation, O-methylation, and acylation [18].

In sum, flavonoids are molecules with a series of beneficial effects, exerting anti-inflammatory [6,9], antioxidant [9], pro-apoptotic [19], anti-aging [1,20], neuroprotective [21], anti-cancer [22], antimicrobial [23,24,25], antiviral [26,27,28], anti-parasitic [28,29], anti-angiogenic [9], cardioprotective [30], and antidiabetic [31] properties.

### Absorption and Metabolism of Flavonoids

Flavonoids are usually present in plants as glycosides, and a hydrolysis enzymatic reaction is required for them to be absorbed as aglycons [32]. The natural glycosylated complexes are firstly de-glycosylated in the small and large intestine by two diverse enzymes, the lactase-phlorizin hydrolase (LPH) and the cytosolic β-glucosidase (CBG).

The LPH enzyme is placed in the microvilli membranes of the brush border of the small intestine and hydrolyzes flavonoid mono-glucosides, forming aglycons. Aglycons are characterized by increased lipophilicity and are able to pass the enterocyte cytoplasm membrane by passive diffusion. Alternatively, the glucosides can be taken up into the enterocytes by specific membrane transporters such as sodium-glucose co-transporter type 1 (SGLT1) and after hydrolyzation by cytosolic β-glucosidase.

Following absorption by intestinal epithelial cells, flavonoid aglycons are mainly conjugated to glucuronic acid or sulfonate group by phase II enzymes, such as uridine-5ʹ-diphosphate-glucuronosyltransferases (UGT) and sulfotransferases (SULT), respectively [32].

These products, through portal circulation, are further metabolized in the liver and conjugated by phase II metabolism enzymes. This leads to the consequent generation of most hydrophilic forms of flavonoids released in the bloodstream that are mainly removed by the renal system.

Some flavonoid metabolites could be excreted via bile in the intestine, where gut microbiota are able to de-conjugate these products that can be further re-absorbed (enterohepatic recycling) [33]. This process enhances the flavonoid half-life in plasma (Figure 2).

Despite the wide class of flavonoids showing bioactive properties, this review is principally focused on the biological properties of luteolin compound and its glucoside form, LUT-7G.

## 2. Luteolin and Its Glucoside LUT-7G

### Luteolin Structure and Natural Plant Sources

Luteolin is a metabolite belonging to the group of flavones. Moreover, this plant-derived compound is characterized by a C6-C3-C6 structure holding two benzene rings (the rings named A and B shown in Figure 1) and one oxygen-containing ring with a 2-3 carbon double bond (the ring named C shown in Figure 1). Luteolin shows hydroxyl groups on 3′,4′,5′,7′ carbons (Figure 1 red), and this chemical configuration, together with the presence of a 2-3 carbon double bond, has been shown to be responsible for its biochemical and biological properties as having anti-oxidant activity [1,9].

The glycoside form (LUT-7-O-glucoside or LUT-7G) is the most common luteolin compound introduced by a diet rich in plant-derived foods and beverages such as cruciferous and green leafy vegetables (red cabbage, kale, spinach, lettuce), herbs (thyme, rosemary, parsley, oregano), carrots, soybeans, blue potatoes, onions, olive oil, berries, citrus fruits (lemons, oranges), pomegranates, apples, grapes, spices, nuts, dark chocolate, coffee, green tea, and seaweed [9,34].

The main difference between luteolin and LUT-7G is the chemical structure (shown in Figure 3). Moreover, comparing the activity of both, luteolin aglycone seems to show a stronger antioxidant, anti-inflammatory, and anti-diabetic effect with respect to LUT-7G glucoside form [35,36].

## 3. Luteolin and LUT-7G in Inflammation

### 3.1. Specifc Pathways Regulated by the Activity of Luteolin/LUT-7G in Inflammation

Inflammation is a defense mechanism by which immune system cells are recruited in the site of infections or damaged tissue. The inflammation status prevents the spread of pathogens and promotes the tissue repair [37], and it can be short-lived (acute inflammation) or long-lasting (chronic inflammation). The crucial steps of inflammatory response are synthetized in Table 3. Luteolin and LUT-7G have been demonstrated to act at the level of the activation of the inflammatory pathway, as well as being able to enhance the resolution of inflammation [38,39].

Microbial agents and endogenous fragments derived by damaged tissues are recognized by PRRs (pattern-recognition receptors) present on the surface of antigen-presenting cells such as dendritic cells and macrophages. Pattern-recognition receptors are classified in Toll-like receptors (TLRs), nucleotide-binding oligomerization domain-like receptors (NLRs), C-type lectin receptors (CLRs), RIG-1-like receptors (RLRs), cytosolic DNA sensors (CDSs), and formyl peptide receptors (FPRs) (see Table 4) [40].

Moreover, toxic compounds, high concentrations of glucose and fatty acid circulating levels in blood [41,42], alcohol, and metals (fluoride, nickel) [43,44,45] are also able to activate immune cells that secrete pro-inflammatory cytokines (interleukin, TNF, interferons), chemokines, and eicosanoids (leukotrienes, prostaglandins). In particular, the binding of PRR receptors with pathogen-associated molecular patterns (PAMPs) induces the production of three key mediators of acute and chronic inflammatory processes: IL−1β, IL-6, and TNF-α.

**Table 4:** Schematic table showing the localization of PPRs, recognized targets, and the inflammatory pathways activated by them.

PRR Receptors		PAMP Ligands	DAMPs Ligands	Refs.
TLRs (TLR 1-9)	Transmembrane protein in plasma membrane or in endosome	LPS (lipopolysaccharide of bacteria), proteins, nucleic acids, glycans	HSPs, S-100 proteins, histones, DNA, RNA, mtDNA, heparan sulfate, fibrinogen, LMW hyaluronan, syndecans, glypicans	[40]
NLRs	Cytoplasmic sensor	Viral DNA, bacterial DNA, bacterial peptidoglycan	Uric Acid, mROS, Histones, LMW hyaluronan	[40]
CLRs	Transmembrane protein in plasma membrane	Glycans of bacteria, glycans of fungi	F-actin, SAP130	[40]
RLRs	Cytoplasmic sensor	Viral RNA	RNA	[40]
CDSs	Cytosolic DNA sensor	Bacterial and viral DNA	DNA	[40,46]
FPRs	Mitochondrial formyl peptide sensor	Pathogen peptides	Formyl peptide	[40,47]

DAMPs related to nuclear (red), cytosolic (green), mitochondrial (violet), plasma membrane (blue), and extracellular matrix (black) compartments [46].

Luteolin and LUT-7G (see further paragraphs for specific effects) are not only able to block the interaction of ligand (PAMPs) with its receptor (PRRs) but also suppress the downstream activation signals [48,49].

### 3.2. Activation of Inflammatory Pathways

The interaction of PAPMs, interleukin 1β (IL−1β), interleukin 6 (IL-6), and tumor necrosis factor α (TNF-α) with their receptors, TLRs, IL−1βR, IL-6R, and TNFR, respectively [50], leads to the activation of intracellular inflammatory signaling pathways [50,51], among them MAPK (mitogen-activated protein kinase), NF-κB (nuclear factor kappa-B), and JAK/STAT (Janus kinase/signal transducer and activator of transcription) pathways [50] (Table 5).

NF-κB is constituted by inducible transcription factors NF-κB1, NF-κB2, RelA (p65), RelB, and c-Rel. In normal conditions, these members are sequestered in the cytoplasm by IκB inhibitory proteins. Primary stimuli such as interactions ligand-receptor TNF/TNFR, IL-1/IL1R, and microbial peptide/TLR trigger the phosphorylation of NF-κB by IKK (IkB kinase), the subsequent NF-κB nuclear translocation and pro-inflammatory gene transcription [50,52].

MAPK is a family of serine-threonine kinases involved in different physiological processes such as proliferation, differentiation migration, apoptosis, and in the initiation of the inflammatory response. MAPK pathways include MAPK, MAPKK (MAPK kinase), and MAPKKK (MAPKK kinase). Primary stimuli such as TNF, IL-1, and IL-6 lead to the phosphorylation of MAPKK by MAPKKK, which in turn phosphorylates and activates MAPK. MAPK finally induces the activation of transcription factors enhancing the inflammatory response [50,53].

The JAK/STAT pathway is commonly involved in immune-mediated diseases [54]. The IL-6 binding with its tyrosine kinase receptor activates the receptor-associated JAKs, which in turn phosphorylate STAT. Phosphorylated STAT can form stable dimers and subsequently translocate from the cytoplasm to the nucleus, activating the transcription of downstream cytokines [50].

Both luteolin and LUT-7G have been demonstrated to downregulate IL−1β, IL-6, and TNF-α, directly counteracting NF-κB, MAPK, and JAK/STAT inflammatory pathways by reducing the inflammation status [9,55,56] in cellular model of inflammation.

NF-kB transcription factor modulates even the expression of inflammatory mediators such as COX-2 (cyclooxygenase 2) and iNOS (inducible nitric oxide synthase). Both luteolin and LUT-7G can counteract this effect by reducing the production of oxide (NO) and prostaglandin E2 more efficiently than LUT-7G. The latter is more efficient in inhibiting the phosphorylation of p65 by blocking its translocation from the cytoplasm into the nucleus [36]. Moreover, luteolin reduces the activation of NF-κB and AP-1, while LUT-7G only repress NF-kappaB activation. Indeed, both flavonoids inhibit Akt phosphorylation in a dose-dependent manner.

Moreover, it has been demonstrated in HEKn cells that LUT-7G treatment is able to impair the nuclear translocation of phosphorylated STAT3 induced by IL-22 and IL-6 stimuli [57]. The treatment of human keratinocytes with LUT-7G can induce a differentiative stimulus counteracting the pro-proliferative action of both cytokines. In vivo experiments performed using a psoriatic mouse model showed that the local treatment with LUT-7G is able to revert the psoriatic phenotype, suggesting another anti-inflammatory use for this flavone.

Overall, both these flavones are able to exert anti-inflammatory activities by inhibiting the production of pro-inflammatory cytokines (IL−1β, IL-2, IL-6, IL-8, IL-12, IL-17, TNF-α, INF-β) [9,51,55], chemokines and their receptors (CCL1, CCL2, CCL3, CCL19, CCL21, CCR7, CCR8, CXCL2, CXCL8, CXCL9, CXCL12) [9,51], prostaglandin E-2, leukotriene C4, COX-2 (cyclooxygenase enzyme), and iNOS (inducible oxide nitric synthetase) [51] (Figure 4, Table 6).

Investigating the role of the two molecules in inflammation, a role for the LUT-7G in reducing inflammation by increasing the level of anti-inflammatory cytokine IL-10 and its receptor, IL10-RB [9,63], also emerged. Moreover, LUT-7G treatment enhances ICEBERG level (caspase-1 inhibitor) by blocking the secretion of IL−1β [64] (Figure 5), as well as inhibiting the NLRP3 inflammasome activation, which is responsible for IL−1β production [65,66] (Figure 5).

The NLRP3 inflammasome is a complex acting as mediator in the innate immune response against bacteria, viruses, and fungi. Moreover, its dysregulation may also be implicated in the pathogenesis of several diseases such as diabetes, atherosclerosis, Alzheimer’s disease, and auto-inflammatory diseases [67].

NLRP3 inflammasome is constituted by the oligomerization of NLRP3 adaptor proteins that interact with ASC (apoptosis-associated speck-like protein containing a caspase-recruitment domain), a protein required for the recruitment of pro-caspase-1 to the NLRP3 inflammasome complex [67]. This proximity enhances the autocatalytic activation of caspase-1 (Casp1), leading to the cleavage of pro-interleukin IL−1β and IL-18 into their active forms. This process increases the inflammatory response. Beyond to the microbial stimuli, NLRP3 inflammasome assembly is enhanced even by the TLR-mediated NF-κB activation signaling via the upregulation of NLRP3 and the synthesis of pro-IL−1β [67].

This cascade of pro-inflammatory events could be reverted by luteolin treatment in vitro and in vivo models since the flavone significantly reduces the cleavage of pro-caspase-1 and pro-IL−1β by NLRP3 inflammasome in murine macrophages. Furthermore, in the murine model, this natural phenol is able to decrease ASC and Casp1 transcription by interfering with priming signals required for the activation of NLRP3 inflammasome [65] (Figure 5).

Luteolin treatment reduces the serum concentration of IL-1β, IL-6, and TNF-α and upregulates FOXP3, which may be related to an increase of Tregs (regulatory T cells) in lung of a murine model. Tregs produce IL-10, an anti-inflammatory cytokine able to suppress inflammatory response via promoting the polarization of M2 macrophages [38,63].

In vivo experiments have reported the systemic effects of luteolin on the brain, liver, and bowel districts. Since luteolin shows anti-inflammatory properties and crosses the blood–brain barrier, it could be a therapeutic candidate for Alzheimer’s disease (AD) [68]. AD is characterized by the accumulation of beta-amyloid proteins, neurofibrillary tangles, oxidative stress, alterations in glucose/lipid metabolism, and inflammation. Overall, luteolin reduces hippocampal inflammation by inhibiting endoplasmic reticulum stress in astrocytes, required for preserving the function of nervous system. This mechanism triggers an improvement of spatial learning and ameliorates memory deficit in the AD rat [21,68].

Recent evidence has suggested that luteolin anti-oxidant and anti-inflammatory properties could counteract the liver damage caused by carbon tetrachloride [69] and metal exposure as lead acetate [70]. This flavone reverts the hepatotoxic condition characterized by oxidant production; causes depletion of antioxidant proteins; increases hepatic enzymes (AST, ALT), total bilirubin, and pro-inflammatory cytokines; and causes the activation of the NF-κB pathway, as well as the apoptotic program [70].

Moreover, luteolin attenuates the liver injury induced by mercuric chloride. This metal causes ROS production and oxidative stress with subsequent activation of NF-κB and TNF-α pathway, leading to hepatocyte apoptosis. Luteolin exhibits anti-inflammatory properties acting on both these pathways [71].

The related benefits of luteolin on the inflammatory process have been extended even to the bowel district. Luteolin could ameliorate the colonic damage in rats with ulcerative colitis by reducing the expression of pro-inflammatory cytokines such as IL-6 and IL-23 and by inhibiting the NF-κB pathway, which is responsible for inflammatory cell infiltration, hyperemia, edema, and ulceration [72]. Moreover, the luteolin administration modulates the gut microbiota composition by promoting microbial diversity as the growth of *Butyricicoccus* [72].

### 3.3. Metabolism and Energy Production

LUT-7G is also able to affect energy production, lipid metabolism, and glucose homeostasis. The metabolomic analysis performed on human keratinocytes has suggested that LUT-7G treatment (20 µM in culture medium) modulates different metabolic pathways such as glycolysis, Krebs cycle, and pentose phosphate pathway.

In particular, LUT-7G significantly reduces intermediate metabolites such as glucose-6-phosphate (G6P) and fructose-6-phosphate (F6P) in the early steps of glycolysis and 3-phosphoglicerate (3PG) and phosphoenolpyruvate (PEP) in the last steps. This could be partially explained by the high affinity of LUT-7G for HEK2 (hexokinase 2) catalytic sites, a key enzyme in the phosphorylation of glucose in G6P. Overall, this underlines the ability of LUT-7G in decreasing ATP synthesis and energy production by blocking the glycolysis pathway [73]. The affinity of the glycosylated flavone is specific since the same modelling using the aglycone showed a lower free-energy binding. This because of the presence of the glucose group that confers the higher affinity with the active site of HEK2 [73].

Moreover, LUT-7G may affect the Krebs cycle by downregulating the intermediates such as citrate, succinate, and fumarate, as well as sedoheptulose-7-P and xylulose-5P metabolites in the pentose phosphate pathway [73].

On the other hand, this flavonoid upregulates a series of vitamins such as cobalamin (vitamin B12), pyridoxin (vitamin B6, coenzyme of glycogen phosphorylase), riboflavin (vitamin B2, component of NAD and FAD, cofactors for flavoprotein enzymes), and thiamin (vitamin B1) [73]. In Table 7, all metabolic pathways affected by the LUT-7G treatment have been reported.

### 3.4. Lipid Pathways Involved in Inflammation

Studies on LUT-7G and lipid profile modification (Table 8) demonstrated that this form of the flavone affects the cholesterol hydroxylation pathway, increasing the cholesterol levels and reducing the 7-alpha hydroxycholesterol, 7-beta hydroxycholesterol, and 7-ketocholesterol levels in the human keratinocyte model [9]. These hydroxylation products, oxysterols, are 27 carbon atom molecules and could be formed by auto-oxidation (mainly 7-ketocholesterol) or cholesterol enzymatic oxidation [74].

Oxysterols exert cytotoxic effects by inducing oxidative stress and disfunction of organelles such as mitochondria, lysosomes, and peroxisomes leading to cell death [74,75]. The increase of cholesterol hydroxylation products has been found in many pathological conditions such as inflammatory bowel diseases, macular degeneration, neurodegenerative disorders (Alzheimer’s disease), and cardiovascular diseases. In fact, oxysterols accumulate in the atherosclerotic plaque and in oxidized low-density lipoproteins (LDL), enhancing the inflammatory processes and artery damage. Moreover, these hydroxylation products stimulate macrophages in secreting pro-inflammatory IL-8 and MCP-1 chemokines, both pro-atherogenic factors [76]. Furthermore, 7-ketocholesterol directly has effects on vascular smooth muscle cells (VSMC) promoting vascular inflammation response through the extracellular secretion of IL-6 [9].

Moreover, LUT-7G even regulates the fatty acid hydroxylation pathway, leading to an increase of linoleic acid levels and a decrease of 2-hydroxypalmitate, 2-hydroxystearate, and 2-hydroxydecanoate levels [9].

These effects on lipid metabolism suggest a strong antioxidant function of LUT-7G compound.

### 3.5. Glucose Homeostasis

Available evidence strongly suggests that type 2 diabetes is an inflammatory disease, and that inflammation is a primary cause of obesity-linked insulin resistance, hyperglycemia, and diabetes [77].

Diabetes is the most common metabolic disorder, characterized by hyperglycemia, insulin resistance (type 2), or insulin deficiency (type 1) associated with alterations of glucose, lipid, and protein homeostasis. In fact, the excessive amount of glucose circulating in the blood causes the glycosylation of many proteins, leading to inflammation and oxidative stress. Flavonoids can significantly regulate the blood glucose balance, counteracting the hyperglycemic state [78]. In this context, all flavonoids could improve insulin secretion by pancreatic cells, as well as insulin sensitivity. Among them, both luteolin and LUT-7G exert antidiabetic activities [31,79]. Despite this, luteolin showed stronger effects than its glucoside LUT-7G in the regulation of blood glucose [35], since luteolin activates eNOS (endothelial nitric-oxide synthetase), restoring the NOS pathway that is generally downregulated in human diabetes as well as in animal experimental models [31]. Moreover, this compound improves the SOD activity required for balancing ROS production [31] and also modulating Akt2 activity by preventing the dephosphorylation of insulin receptor (IR). Phosphorylated IR maintains active insulin signaling, and therefore glucose uptake [31]. Likewise, luteolin enhances SIRT-1-SIRT-3-SIRT-6 and FOXO3a expression, inhibiting the high glucose-induced ROS production in human monocyte cells [79]. Since this compound is able to cross the blood–brain barrier, it can directly modulate the secretion of glucagon-like peptide 1 (GLP-1) by the hypothalamus, which is involved in the regulation of energy homeostasis through the suppression of appetite [80].

### 3.6. Anti-Inflammatory Properties Connected to the Anti-Oxidant Activity of Luteolin

Oxidative stress can lead to chronic inflammation [81], a condition that occurs in cells characterized by a disequilibrium between the levels of reactive oxygen species (ROS) and anti-oxidant defense (enzymes such as SOD, catalase, GST, glutathione, vitamin C, vitamin E, and carotenoids) [82,83]. ROS production is mainly due to the electron transfer from the oxygen along the enzymes of the respiratory chain. If not balanced, ROS could induce DNA damage leading to mutagenesis and cancer, protein cross-linking, lipid peroxidation of poly-unsaturated fatty acids causing cellular aging, impairment of cell membranes, and the oxidation of low-density lipoproteins with consequent vascular system damage [84].

Luteolin shows a particular chemical structure responsible for its antioxidant and anti-inflammatory activity. The 2-3 carbon double bond of C ring (C2=C3) conjugated with a carbonyl group in C4 is able to chelate Fe^2+^ ions (metal chelating capability), required as a catalyst in the Fenton reaction to produce OH· radicals (hydroxyl radical, see Figure 6), the reactive oxygen species most likely to cause lipid oxidation [73,85].

Moreover, the addition of the sugar group in the luteolin glycosylated form (LUT-7G) makes the compounds more polar, increasing its antioxidant activity and therefore inhibiting lipid peroxidation [86]. For this reason, both luteolin and LUT-7G can be considered free radical scavengers, with an increased activity of the glycosylated form.

Luteolin antioxidant properties have been tested in vitro and in vivo models; in NRK-52E rat kidney cells incubated with ochratoxin A (OTA), a ubiquitous carcinogen food contaminant, luteolin treatment counteracts the OTA nephrotoxic effects by restoring the kidney cell antioxidant capability through Nrf2 activation [87]. Luteolin-mediated Nrf2 activity confers even renoprotection against ischemia-reperfusion injury [88] and alleviates bisphenol A-induced nephrotoxicity in rat models. In this context, luteolin shows a double effect as it reduces the lipid peroxidation linked to the production of pro-inflammatory lipids, as well as DNA damage by upregulating HO-1 (heme oxygenase) alongside Nrf2, both required against oxidative stress [89]. LUT-7G has been demonstrated to also act as a ROS scavenger in different cellular systems [57,73]. Moreover, an important antioxidant function of LUT-7G glucoside on the cardiovascular system is discussed in a further specific paragraph [9].

## 4. Anti-Aging Properties of Luteolin

Recent studies have reported the anti-aging property of luteolin on rat skin models [20]. The skin aging is mainly due to solar ultraviolet radiation exposure, which induces inflammation; the formation of pyrimidine dimers (DNA damage); and oxidative stress that affects lipids, proteins, mitochondria, and DNA. These actions lead to the onset of immediate and long-term effects such as sunburn, elastin and collagen fiber degradation, and the wrinkled appearance known as photoaging. UV induces the production of hydroxyl radicals (reactive oxygen species, ROS) able to interfere with cell structures. ROS activate AP1, NF-kB, and MAPK pathways and the following transcription of downstream genes such as COX-2 and PGE_2_ [1].

Luteolin exerts protective functions by absorbing a large amount of solar UV radiation, reducing the UV amount transmitted to the cell, and by stabilizing with its C2-C3 double, the radical species preventing the oxidative damage. This function impairs the MAPK signaling pathway and COX-2 and PGE_2_ synthesis [1].

On the other hand, luteolin could stabilize mitochondrial function, restoring SIRT-3 expression, downregulated by oxidative stress [20]. In fact, SIRT-3 (NAD^+^-dependent lysine deacetylase) avoids ROS accumulation in the mitochondria by deacetylating mitochondrial enzymes that show increased ROS scavenging activity.

## 5. Anticancer Activity of Luteolin/LUT-7G

Beyond antioxidant and anti-inflammatory effects, luteolin shows anti-cancer activity by also modulating glucose metabolism, cell growth pathways, and factors involved in the apoptosis process.

Malignant cells acquire the ability to reprogram their metabolism from oxidative phosphorylation to aerobic glycolysis, which allows for the support of rapid cell division. In normoxic conditions, physiological cells produce ATP by oxidative phosphorylation, while cancer cells transform pyruvate in lactate through lactate dehydrogenase A (LDHA). This metabolic switch is known as the “Warburg effect” and it is orchestrated by the dysregulation of many factors, among these, glucose transporters (GLUT), hypoxia-inducible factors (HIFs), pyruvate kinase muscle isoform 2 (PKM2), and hexokinase 2 (HEK2) metabolic enzymes [90]. Flavonoids are able to modulate the key factors involved in the Warburg effect—luteolin-O-β-D-glucoside reduces LDH enzyme activity [91], quercetin and apigenin reduce the glucose uptake suppressing the activity and expression of GLUT-1/4, and resveratrol decreases HIF-1 levels, suggesting a crucial role for all flavonoids in the carcinogenesis process [90].

Moreover, luteolin treatment exerts the following effects on JAK/STAT, wnt/β-catenin, and Notch signaling:

- The JAK/STAT signaling pathway plays opposite roles in the carcinogenesis process depending on the ligand/receptor and member of the STAT (STAT1, STAT2, STAT3, STAT4) family involved. In the IFN alpha/beta (ligand)-IFNRA1-2 (receptor) activation pathway, luteolin is able to maintain the phosphorylated status of STAT1 by inhibiting the SHP-2 dephosphorylase. In this context, activated STAT1 signaling arrests the cancer growth. In contrast, luteolin can reduce the phosphorylated levels of STAT3, leading to tumor suppression in breast cancer since STAT3 is a transcription factor for S100 calcium-binding protein A7 (S100A7) required in the metastasis formation [57,92].
- Tumor cells frequently report a dysregulation of wnt/β-catenin and Notch signaling, leading to EMT transition and metastasis. Luteolin is able to modulate both these pathways by downregulating β-catenin and Notch-1 [92].

Luteolin can also exert a pro-apoptotic activity, directly suppressing the BCL-2 anti-apoptotic protein, which is overexpressed in SW1990 pancreatic cancer cells [93].

In A549 human lung cancer cells, luteolin downregulates Rac1, RhoA, and cdc42, leading to a decrease in cell viability, filopodia formation, migration, and invasion [22]. Moreover, luteolin acts as a tumor suppressor by upregulating p53 and p21 pro-apoptotic proteins in both A549 and H460 cell lines [94], suggesting the anti-cancer ability for this flavone.

## 6. The Role of Luteolin on Vascular Function

**Figure 7:**
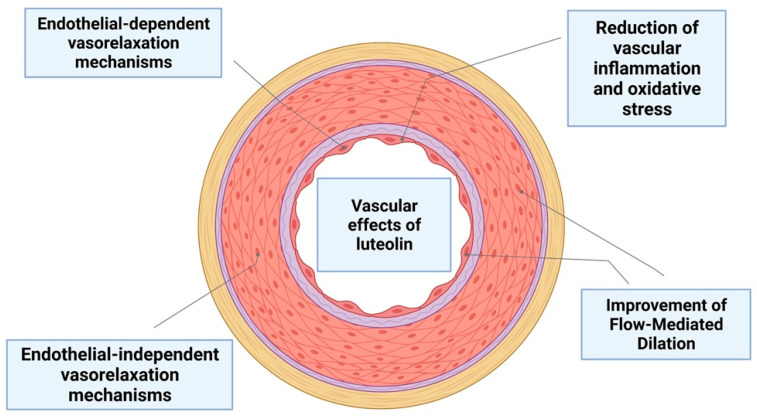
Luteolin and LUT-7 effects on vascular physiology.

**Figure 1:**
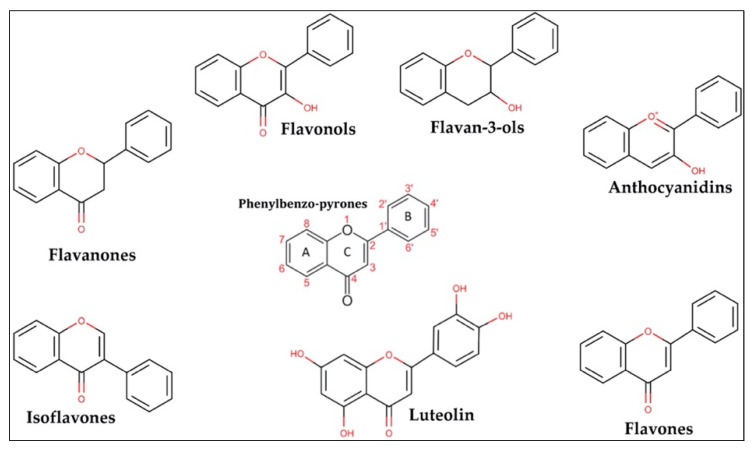
Chemical structures of flavonoid subclasses [9].

**Figure 2:**
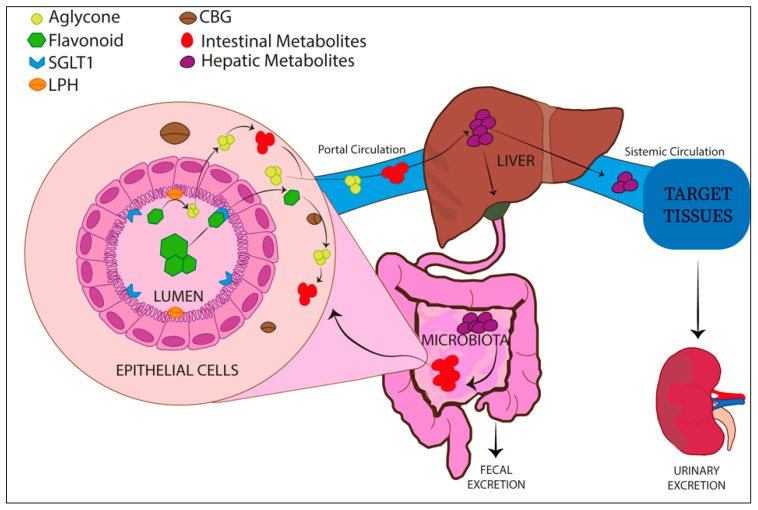
Schematic overview of flavonoid metabolism, showing the uptake and the modifications of the flavone in the different districts/organs. Sodium-glucose co-transporter type 1 (SGLT1), cytosolic β-glucosidase (CBG), lactase-phlorizin hydrolase (LPH).

**Figure 3:**
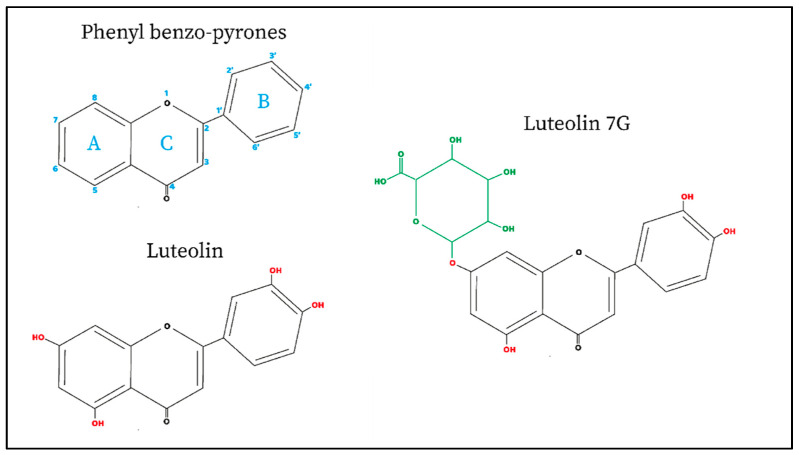
Basic chemical structure and molecular geometry of phenyl benzo-pyrones, luteolin (3′,4′,5′,7′-tetrahydroxy flavone), and LUT-7G.

**Figure 4:**
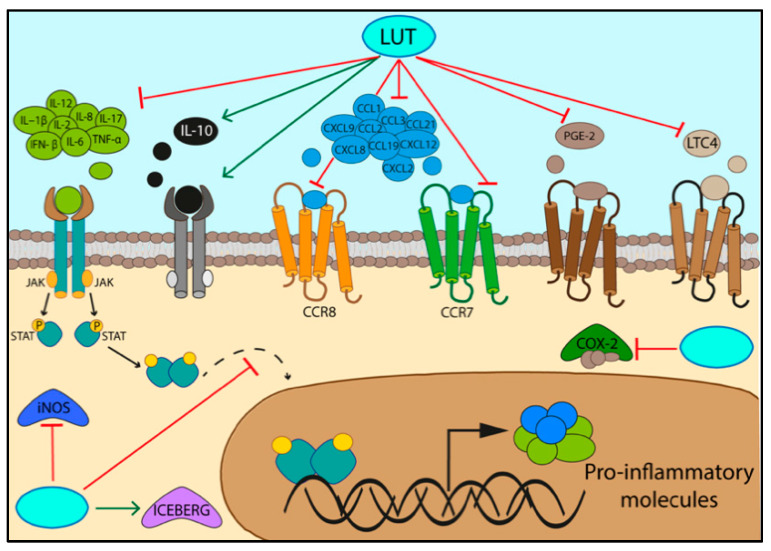
Luteolin and LUT-7G anti-inflammatory properties.

**Figure 5:**
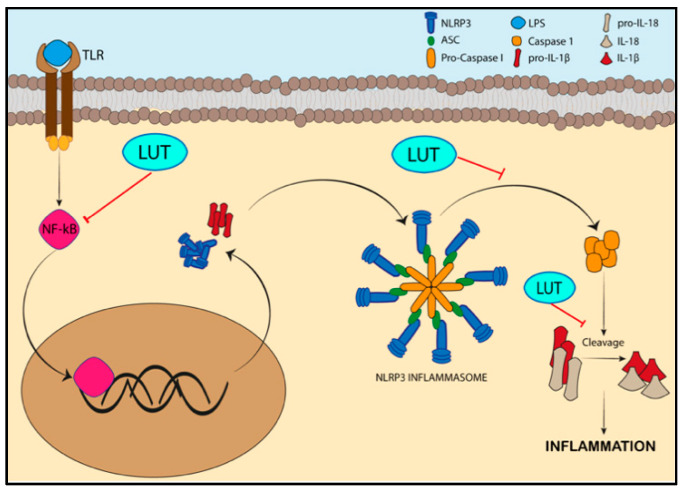
Luteolin effects on the NLRP3 inflammasome. TLR-mediated NF-κb activation induces the transcription of NLRP3 required for NLRP3 inflammasome assembly and pro-IL-1β. Luteolin could interfere with NF-κb, blocking the upstream process involved in the inflammasome recruitment. Moreover, LUT decreases the cleavage of pro-caspase-1 and pro-IL−1β, a downstream event of NLRP3 inflammasome activation.

**Figure 6:**
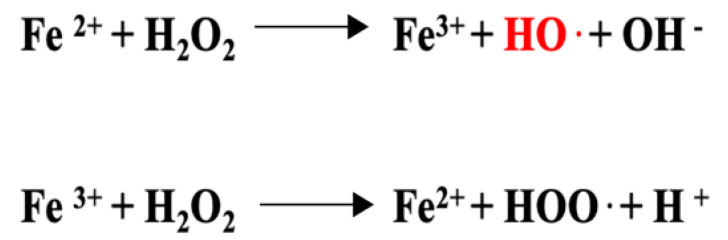
Fenton reaction uses the hydrogen peroxide and catalytic ferrous (Fe^2+^) ion to produce the hydroxyl radical. Luteolin exerts the metal (Fe^2+^) chelating capability, avoiding the formation of OH·, the most dangerous ROS for cells. H_2_O_2_ = hydrogen peroxide, OH· = hydroxyl radical, OH^−^ = hydroxyl, HOO· = hydroperoxyl, H^+^ = proton.

The role of luteolin and LUT-7G on vascular function has been elucidated and confirmed by several pieces of evidence, as well as its potential anti-remodeling and antihypertensive properties. Results from experiments in vivo in SHR mice (spontaneously hypertension mice model) have suggested that luteolin is able to lower arterial blood pressure as well the media thickness of vessel walls, moreover reducing hypertensive remodeling in the vasculature. This effect of luteolin is in part related to inhibition of either vascular smooth muscle cell (VSMC) proliferation or migration, due to the inhibition of RAAS (renin–angiotensin–aldosterone system) pathway activation, angiotensin II expression, ROS production, and the activation of the MAPK pathway [95], (Table 9, Figure 7).

Cardiomyocyte function is regulated by MAPKs. It was proposed that luteolin is able to improve cardiomyocyte function by regulating MAPK proteins. Furthermore, luteolin was found to be capable of regulating nitric oxide (NO) and NO synthases to prevent oxidative stress and inflammation in coronary artery diseases (CAD) [30]. Atherosclerosis is a vascular disease of the coronary arteries and the peripheral and cerebrovascular vasculature. The hallmarks of atherosclerosis include accumulation of lipids, cell proliferation and migration, inflammation, and NO reduction that leads to endothelial dysfunction [96]. The anti-inflammatory effects of luteolin as well as its inhibition properties on both cell proliferation and migration were reported in several studies. The activation of the JAK/STAT3 pathway is a pivotal mechanism involved in the endothelial inflammation in the vasculature. Experiments in endothelial cells (HUVEC) exposed to luteolin have investigated its effects on the inhibition of STAT3. These results suggest the hypothesis that luteolin antagonizes the pro-proliferative and pro-inflammatory properties of STAT3. Moreover, the analysis of Ki67, a specific marker of proliferation, has shown the decrease of the endothelial cell proliferation after exposure to luteolin. Endothelial cells are also involved in the synthesis of the von Willebrand factor (vWF) [97]. A consistent cytoplasmic location reduction of vWF was found in treated cells with luteolin, thus confirming the pro-differentiating and antiproliferative properties of luteolin on endothelial cells [9]. Recently, studies conducted ex vivo showed that the luteolin induces an endothelium-independent vasorelaxation in uterine arteries in pregnant mice that is mediated by the medial smooth muscle layer [98].

In contrast, luteolin evoked a vasoactive property by directly acting on vascular endothelial cells to stimulate NO-dependent vascular relaxation in rat aortic rings [99].

In an in vivo study on high-fat diet (HFD) mice, luteolin showed anti-obesity properties, lowering the body and epididymal fat weight, as well as metabolic obesity-related complications, including vascular dysfunction. Furthermore, luteolin was reactivated in terms of vascular endothelial NO availability, inhibiting the ROS and TNF-α (tumor-necrosis factor alpha) action [100]. The effect of supplementation of luteolin on the cardiometabolic risk factor has also been evaluated in human clinical trials. In a randomized, double-blind, placebo-controlled study, 100 participants were enrolled with metabolic syndrome (MetS) during a 6 month period follow-up; 50 patients were allocated randomly to receive Altilix^®^ and the other 50 to a placebo. Cardiometabolic parameters were measured, and the thickness of carotid intima-media and endothelial function were analyzed by doppler ultrasound and by flow-mediated dilation of the brachial artery. After 6 months of Altilix^®^ administration, the researchers found a great improvement of cardiometabolic parameters such as body weight, waist circumference, and HbA1c (glycated hemoglobin) and plasma lipids in treated subjects compared to the placebo. An improvement of vascular function by flow-mediated dilation and carotid intima-media thickness was also demonstrated [101].

**Table 9:** Specific effects of treatment with luteolin and LUT-7G on vascular physiology.

Molecular Mechanisms	Treatment	Cell Lines/Animal Model/Humans	Refs.
Antihypertensive effects by inhibition of RAAS	LUT	SHR mice	[95]
Anti-inflammatory effects by inhibition of JAK/STAT3 pathway	LUT-7G	Human endothelial cells (HUVEC	[9]
Endothelium-independent vasorelaxation	LUT	Pregnant rats (uterine arteries)	[98]
Endothelium-dependent vasorelaxation	LUT	Rat aortic rings	[99]
Inhibition the ROS and TNF-α action and improvement of vasodilation	LUT	HFD mice	[100]
Improvement of flow-mediated dilation of the brachial artery	Altilix	Humans	[101]

## 7. Discussion

Taken together, plant-derived food and fruits rich in oligosaccharides and fibers provide a major source of polyphenols, whose benefits by improving individual health have been fully accepted in the scientific literature. In the specific case of luteolin and its glycoside form, the overall scientific data regarding their biological effects suggests an impacting role for these natural compounds by negatively modulating pro-inflammatory and pro-oxidant signaling. Indeed, chronic inflammation, ROS production, and lipid/protein oxidation have been found in the onset of several human diseases such as psoriasis; atherosclerosis; cardiovascular, metabolic, and neurodegenerative disorders; and even in aging [1,9,21]. Luteolin and its glycoside have been described to regulate NF-κB, MAPK, and JAK/STAT pathways, modifying the effects induced by different pro-inflammatory cytokines such as TLRs, TNF, IL-1TNF, IL-1, and IL-6. Connected with these well-characterized properties, recent studies have been demonstrated the ability of luteolin in modulating glucose metabolism, cell growth, and the apoptosis process, which are frequently dysregulated in malignant cells. This evidence could suggest a role of luteolin as an anti-cancer compound [90], considering that chronic inflammation is also important in this pathology. Moreover, besides the effects on glucose metabolism, luteolin may represent a potential treatment for diabetes, and it might even ameliorate the glucose uptake via restoring the insulin signaling in an insulin-resistance background [31].

It is important to note that the presence of a glucose group in LUT-7G confers different chemical properties to the molecule. A wide number of specific effects on the molecular players of the inflammation processes are retained by both molecules, such as the action on JAK/STAT3 or NF-κB pathways, while others, such as the affinity for HEK2, are specific for LUT-7G [73]. Moreover, the presence of the glucose confers hydrophilicity to the compound, as well as the possibility of using GLUT transporters. On the other hand, the high lipophilicity of luteolin alone provides the possibility of easily crossing the plasma membrane. These properties should be taken in account for possible different use of the two molecules, considering also recent data showing the capacity of luteolin to induce hormetic dose responses [102].

The different capabilities and multi-organ protective properties attributable to the anti-inflammatory properties of luteolin molecules as well as other flavonoids propose the high relevance of introducing plant-derived food to the diet. It is important, however, that these interesting properties cannot be completely achieved by diet supplementation, since a pharmacological concentration is difficult to reach, and does not involve natural extracts that normally contain important contamination of other molecules of unknown activity. Dietary supplementation could be helpful in terms of prevention in order to reduce the oxidation state and the principles of inflammation. On the other hand, the use of these molecules in purified pharmacological preparation could be used to deeply investigate their potential clinical use.

Currently, the molecules are available on the market only as analytical standards. Nevertheless, in an attempt to find in a brief period of time these flavones, even in the pharmacopeia, we herein described their pleiotropic anti-inflammatory action.

## Figures and Tables

**Table 1:** Foods and beverages rich in flavonoid content.

DIETARY SOURCES OF FLAVONOIDS	
**Vegetables**	Broccoli, spinach, red cabbage, onion
**Fruits**	Citrus fruits, blackberries, blueberries, strawberries, raspberries, currants, grapes, plumps, apples, nuts
**Beverages**	Tea, red wine
**Other foods**	Cereals, dark chocolate, spices, soy milk

**Table 2:** Examples of flavonoid compounds divided for each group.

FLAVONOID CLASSIFICATION	
**Flavones**	luteolin, luteolin glucosides, apigenin, chrysin, rutin
**Flavonols**	quercetin, kaempferol, myricetin, tamarixetin
**Flavan-3-ols**	catechin, epicatechin, apigallocathechin gallate
**Flavanones**	naringin, taxifolin, hesperidin, eriodityol, naringenin
**Isoflavones**	genistin, genistein, daidzin, daidzein
**Anthocyanins**	apigenidin, cyanidin

**Table 3:** Classical division of sequential steps of the inflammation process.

Phases of Inflammatory Response
Recognition of microbial and endogenous fragments by cell surface receptors
Activation of inflammatory pathways
Release of inflammatory mediators
Recruitment of immune cells
Removal of harmful stimuli
Initiation of tissue repair
Resolution of inflammation

**Table 5:** Intracellular inflammatory signaling pathways.

Inflammatory Pathways	Primary Stimuli
NF-κB	TLRs, TNF, IL-1
MAPK	TNF, IL-1, IL-6
JAK/STAT	IL-6

**Table 6:** Summary of luteolin and LUT-7G effects in regulating pro-inflammatory and anti-inflammatory mediators.

**Downregulated Target**	**Treatment**	**Cell Lines/Animal Model**	**Refs.**
IL−1β	LUT	Rat chondrocites	[58]
TNF-α, COX-2, iNOS	LUT	Mouse alveolar macrophages (MH-S)Mouse macrophages (RAW 264.7)	[59]
IL-6	LUT	Murine model	[60]
IL-8	LUT	Human retinal pigment epithelial cells (h-RPE)	[61]
IL-2, IL-12, CXCL9, IL-17, CXCL2, CXCL8,	LUT	Bone marrow-derived macrophages	[51]
PGE2, INF- β	LUT	Mouse macrophages RAW 264.7	[51]
CCL1, CCL2, CCL3, CCR7 CCL19, CCL21, CCR8, CXCL12	LUT-7G	Human endothelial cells (HUVEC)	[9]
Leucotriene C4	LUT-7G	Bone marrow-derived mast cells	[62]
**Upregulated Target**	**Treatment**	**Cell Lines/Animal Model**	**Refs.**
IL-10		Murine model	[63]
IL10-RB	LUT-7G	Human endothelial cells (HUVEC)	[9]
ICEBERG level	LUT-7G	Human endothelial cells (HUVEC)	[9]

**Table 7:** Modulation of metabolites and vitamins by LUT-7G treatment on human keratinocytes.

Metabolic Pathways	Effects of LUT-7G Treatment
Glycolysis	G6P (↓), F6P (↓), 3PG (↓), PEP (↓), Riboflavin (↑), Thiamin (↑)
Krebs Cycle	Succinate (↓), Fumarate (↓), Riboflavin (↑)
Pentose Phosphate	Sedoheptulose-7-P (↓), Xylulose-5P (↓), Riboflavin (↑)
Oxidative Phosphorylation	Riboflavin (↑)
Glycogenolysis	Pyridoxin (↑)
Lipid Metabolism	Cobalamin (↑), Riboflavin (↑)
Metabolism of Amino Acid	Cobalamin (↑), Riboflavin (↑)
Catabolism of Amino Acid	Thiamin (↑), Riboflavin (↑)

G6P: glucose-6-phosphate, F6P: fructose-6-phosphate, 3PG: 3-phosphoglicerate, PEP: phosphoenolpyruvate. (↓): decrease of metabolites/vitamins after LUT-7G treatment. (↑): increase of metabolites/vitamins after LUT-7G treatment.

**Table 8:** Effects of LUT-7G (20 µM) on lipid profile.

LUT-7G EFFECTS ON LIPID PROFILE		
Cholesterol hydroxylation pathway	Cholesterol	↑
	7-Alpha hydroxycholesterol (oxysterol)	↓
	7-Beta hydroxycholesterol (oxysterol)	↓
	7-Ketocholesterol (oxysterol)	↓
Fatty acid hydroxylation pathway	Linoleic acid	↑
	2-Hydroxypalmitate	↓
	2-Hydroxystearate	↓
	2-Hydroxydecanoate	↓

Metabolomic analysis was performed on human keratinocytes [9]. (↓): decrease of lipids after LUT-7G treatment. (↑): increase of lipids after LUT-7G treatment.

## Data Availability

Not applicable.
